# Acute ST segment elevation during exercise stress echocardiography due to severe pulmonary hypertension

**DOI:** 10.1186/1476-7120-9-18

**Published:** 2011-06-06

**Authors:** Tung H Nguyen, Leonardo C Clavijo, Tasneem Z Naqvi

**Affiliations:** 1 Echocardiographic Laboratories, USC University Hospital, Cardiovascular and Thoracic Institute, and LAC+USC Medical Center; Keck School of Medicine, University of Southern California, Los Angeles, California, USA

## Abstract

A 51-year-old female undergoing an outpatient stress echocardiogram to evaluate atypical chest pain developed acute ST elevation in the anterior precordial leads on electrocardiogram following exercise. Echocardiography revealed a severe rise in pulmonary artery systolic pressure (PASP) with marked right ventricular (RV) enlargement and interventricular septum flattening. Subsequently, cardiac catherization confirmed an exercise-induced elevation in PASP and diagnosed pulmonary arterial hypertension without evidence of coronary artery disease. This case suggests that an acute elevation in pulmonary artery pressure with RV dilation may be a potential cause of acute ST elevation during stress testing.

## Case Report

**Figure 1:**
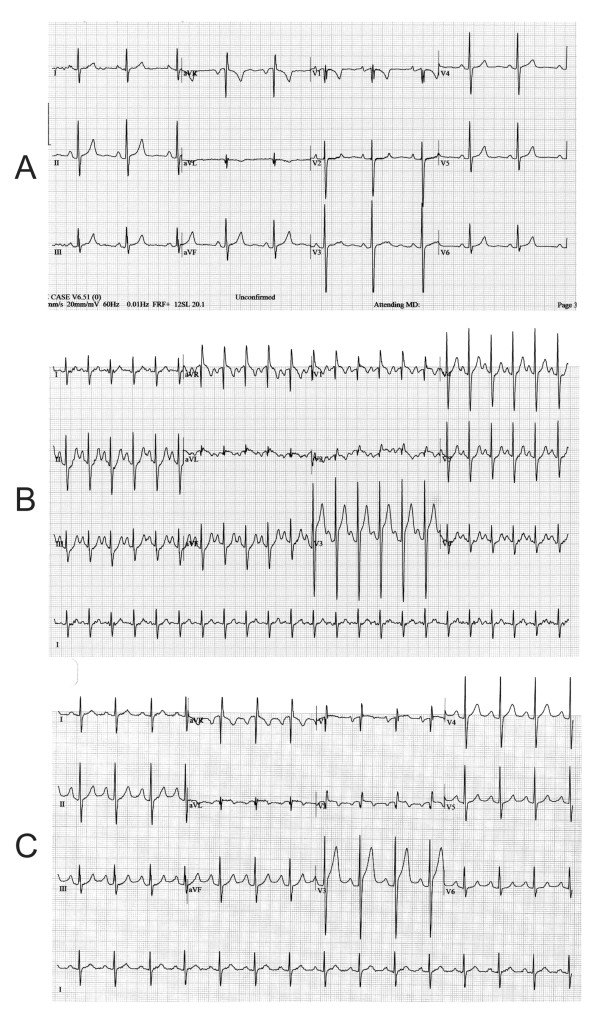
**ECG at rest (A), peak exercise (B), and during recovery (C)**. ECG was recorded at 25 mm/s, 20 mm/mV, and 60 Hz. ST elevation is apparent in leads V1-V3 with exercise, and is most prominent in V3 (B and C). In lead II, the height of the P wave becomes more prominent during exercise and persists into recovery (B and C). Note the deeper S-wave in leads V5 and V6 at peak stress (B) and in leads V5 and lead I during the recovery phase (C).

A 51-year-old female ex-smoker with a history of hypertension, hepatitis C, and HIV on highly active anti retroviral therapy was referred to cardiology clinic for an exercise stress echocardiogram to evaluate atypical chest pain. Resting blood pressure (BP) was 137/97 mmHg. The electrocardiogram (ECG) was notable for a normal sinus rhythm with a rate of 63 beats per minute (bpm), normal axis, and RSR' in V1 with T-wave inversion (Figure 1A). She performed exercise on an exercise bike (Ergometer). Definity contrast was administered at rest and stress for endocardial border definition. Peak heart rate was 141 bpm (83.4% of predicted) and peak BP was 136/100 mmHg. The test was terminated secondary to shortness of breath and leg fatigue. The patient did not experience any chest pain during exercise or recovery. The patient developed up to 2.5 mm acute ST segment elevation in leads V1-V3 during exercise that persisted into the recovery phase (Figure 1B and 1C). RSR pattern became more prominent and extended to leads V1 and V2 (Figure 1B and 1C). Additionally, the height of the P wave in lead II increased during exercise and persisted during recovery. The normal axis at baseline shifted to a rightward axis during the recovery phase, as suggested by more prominent S waves in lead I, V5 and V6 (Figure 1B and 1C).

**Figure 2:**
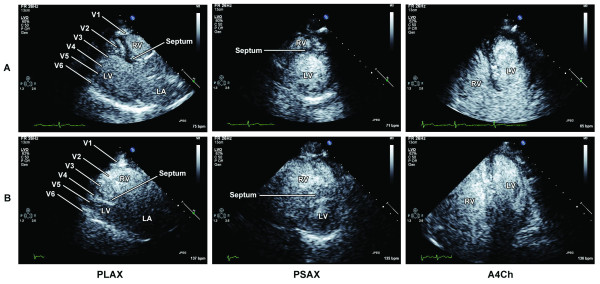
**Parasternal long axis (PLAX), parasternal short axis (PSAX), and apical 4 chamber (A4Ch) views at rest (A) and stress (B)**. In the PLAX view during the stress phase, note the marked right ventricular enlargement into the region typically covered by leads V1-V3.

**Figure 3:**
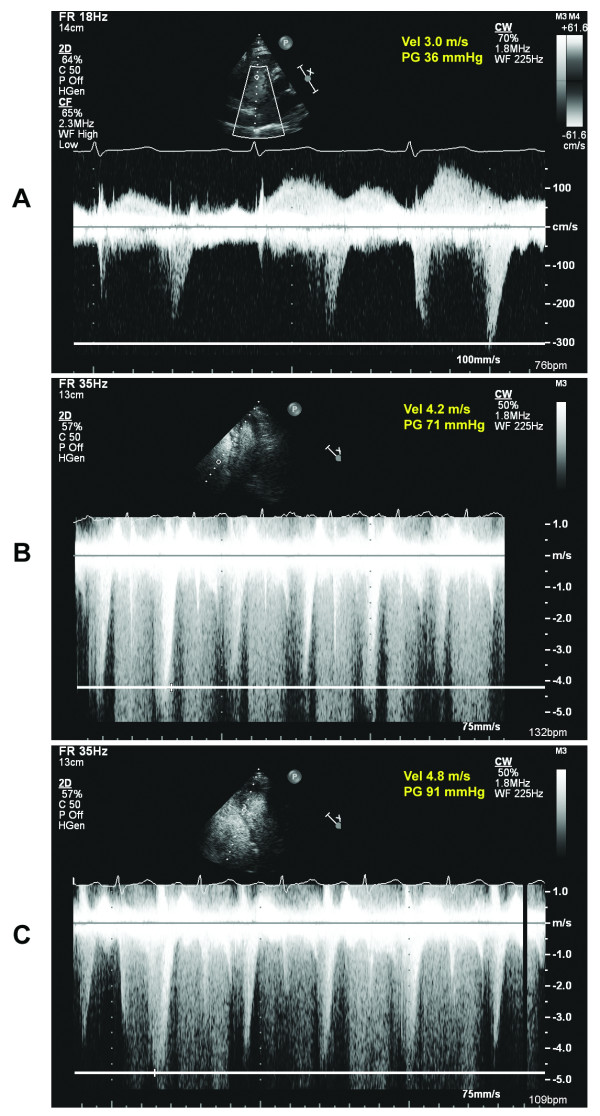
**Continuous wave Doppler across tricuspid valve at rest (A), stress (B) and recovery (C)**. The estimated PASP (ΔP = 4V^2^+RAP, where V is the tricuspid regurgitation velocity and RAP is the estimated right atrial pressure of 10 mmHg) is 46 mmHg at rest, 81 mm Hg during stress and 101 mm Hg immediately post stress.

The resting echocardiogram revealed a normal left ventricle (LV) with an estimated ejection fraction of 65% and wall motion was also normal in all segments (Figure 2A; Additional files 1, 2). There was mild to moderate right ventricular (RV) enlargement and hypertrophy. A saline contrast study did not reveal intracardiac shunting. There was normal valve function. Pulmonary artery systolic pressure (PASP) was estimated at 46 mmHg (Figure 3A). At peak exercise and in the immediate recovery phase, there was marked RV enlargement and interventricular septum flattening (Figure 2B; Additional files 3, 4). The peak PASP was estimated to be 81 mmHg during exercise and 101 mmHg immediately afterwards (Figures 3B and 3C). Wall motion was hyperkinetic in all LV segments and the ejection fraction was 80%. There was no increase in mitral regurgitation during exercise.

**Figure 4:**
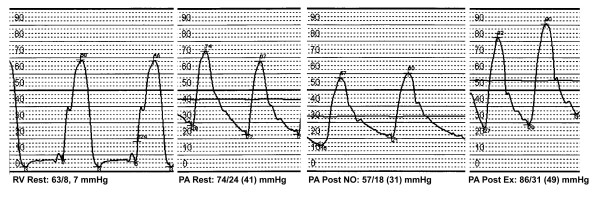
**Right ventricle (RV) pressure at rest, pulmonary artery (PA) pressure at rest, PA pressure after nitric oxide (NO), and PA pressure after exercise**. Note the response to NO.

Seven weeks later, the patient underwent a cardiac catherization. Left heart catherization revealed normal coronary arteries and LV systolic function. There was no coronary vasospasm during the angiogram. On right heart catherization, the resting mean PAP was 41 mmHg and PASP was 74 mmHg (Figure 4). Pulmonary capillary wedge pressure was 7 mmHg. The mean PAP decreased to 31 mmHg following administration of nitric oxide. After exercise, the mean PAP increased to 49 mmHg and PASP was 86 mmHg.

## Discussion

Acute ST elevation during exercise testing is rare with an incidence of 0.78% in those without prior history of myocardial infarction [1] and is most commonly secondary to critical proximal LAD obstruction, LV aneurysm, anterior myocardial infarction [2] and occasionally coronary vasospasm [3]. Our patient demonstrated acute ST segment elevation in the anterior precordial leads with normal coronary arteries on angiography. Mechanism of ST elevation in leads V1-V3 during exercise remains unclear. ST elevation was associated with a marked dilation of RV and development of RV strain pattern on ECG and severe elevation in PASP during exercise. We speculate that sudden pressure load on RV can cause focal or global myocardial ischemia or even injury resulting in ST elevation. Acute RV dilation probably caused RV to displace LV with respect to ECG leads that are fixed on the chest wall. As a result, the anterior precordial leads detected forces that were more prominent from the RV rather than the LV. More prominent right axis deviation as well as increase in P wave height at peak exercise also supports ECG changes secondary to right heart dilation. There was hyperkinetic LV wall motion in all segments which makes vasospasm unlikely and in addition no epicardial artery vasospasm was observed without provocation on coronary angiogram, although we cannot rule out microvascular coronary vasospasm as a cause of ECG changes. Increase in PASP is normally expected during exercise in healthy subjects however peak PASP at exercise does not rise above 43 mm Hg [4]. On RHC, the 10 mmHg decrease in mean PAP following administration of nitric oxide suggests that the pulmonary hypertension was reactive.

ST elevation has been reported in a patient with pulmonary embolism. The mechanism of ST elevation was speculated to be secondary to paradoxical embolization of conus branch of right coronary artery via interatrial shunt in this patient with otherwise normal epicardial coronary arteries [5]. There have been only a few other reported cases of acute RV dilation during exercise stress testing. Acute RV dilation and pulmonary hypertension during exercise was described in a case of pulmonary embolism that was incidentally found on routine stress echocardiogram [6]. Two patients with a history of chronic pulmonary thromboembolism have also been reported to have acute RV dilation following exercise [7]. However, the unique ECG changes associated with RV dilation and an acute severe rise in PAP have not been previously described.

The difference in resting PASP by echo and RHC could be due to underestimation of PASP by resting echo [8] or an interval progression of pulmonary hypertension during seven weeks between the two procedures as pulmonary hypertension secondary to HIV is more rapidly progressive than idiopathic cases [9].

## Conclusion

Although the most likely etiology of ST elevation in patients undergoing exercise ECG testing is coronary artery disease and rarely coronary vasospasm, our case highlights that an acute severe rise in PAP and associated RV dilation may be another possible cause of ST elevation during stress testing. This awareness will help guide further workup and diagnosis in patients manifesting this phenomenon.

## Consent

An attempt was made at contacting the patient to obtain consent but the patient could not be reached.

## Competing interests

The authors declare that they have no competing interests.

## Authors' contributions

THN, LCC, and TZN were involved in writing and revising the manuscript. LCC performed the angiogram and invasive hemodynamic studies. TZN interpreted the stress echocardiogram and picked up the RV enlargement, pulmonary HTN and ST elevation. All authors read and approved the final manuscript.

## Supplementary Material

Additional file 1 **Resting echocardiogram in the parasternal short axis view**. Parasternal short axis at rest showing normal LV wall motion and LV systolic function. The RV is mildly to moderately enlarged and hypertrophied. Click here for file

Additional file 2 **Resting echocardiogram in the apical 4 chamber view**. Apical 4 chamber view at rest showing normal LV wall motion and ejection fraction. The RV is mildly to moderately enlarged and hypertrophied. Click here for file

Additional file 3 **Stress echocardiogram in the parasternal short axis view**. Parasternal short axis during stress showing marked RV dilation with interventricular septum flattening. LV wall motion is hyperkinetic in the mid segments Click here for file

Additional file 4 **Stress echocardiogram in the apical 4 chamber view**. Apical 4 chamber view during stress showing marked RV dilation with interventricular septum flattening. LV wall motion is hyperkinetic in the lateral wall, apex and interventricular septum. Click here for file
